# A predictive model to estimate the pretest probability of metastasis in patients with osteosarcoma

**DOI:** 10.1097/MD.0000000000005909

**Published:** 2017-01-20

**Authors:** Sisheng Wang, Shaoluan Zheng, Kongzu Hu, Heyan Sun, Jinling Zhang, Genxiang Rong, Jie Gao, Nan Ding, Binjie Gui

**Affiliations:** a Department of Joint and Reconstructive Microsurgery, the First Affiliated Hospital of Anhui Medical University, He Fei; b Xia Men Hospital of Traditional Chinese Medicine, Department of Thoracic Surgery, Xia Men, China.

**Keywords:** metastasis, models, osteosarcoma, probability, receiver-operating characteristic curve

## Abstract

Osteosarcomas (OSs) represent a huge challenge to improve the overall survival, especially in metastatic patients. Increasing evidence indicates that both tumor-associated elements but also on host-associated elements are under a remarkable effect on the prognosis of cancer patients, especially systemic inflammatory response. By analyzing a series prognosis of factors, including age, gender, primary tumor size, tumor location, tumor grade, and histological classification, monocyte ratio, and NLR ratio, a clinical predictive model was established by using stepwise logistic regression involved circulating leukocyte to compute the estimated probabilities of metastases for OS patients. The clinical predictive model was described by the following equations: probability of developing metastases = ex/(1 + ex), x = −2.150 +  (1.680 × monocyte ratio) + (1.533 × NLR ratio), where is the base of the natural logarithm, the assignment to each of the 2 variables is 1 if the ratio >1 (otherwise 0). The calculated AUC of the receiver-operating characteristic curve as 0.793 revealed well accuracy of this model (95% CI, 0.740–0.845). The predicted probabilities that we generated with the cross-validation procedure had a similar AUC (0.743; 95% CI, 0.684–0.803). The present model could be used to improve the outcomes of the metastases by developing a predictive model considering circulating leukocyte influence to estimate the pretest probability of developing metastases in patients with OS.

## 1 Introduction

Osteosarcomas (OSs), constituting nearly 1% of newly diagnosed malignancies annually, come from almost any embryonic mesodermal tissues.^[1]^ Estimated 5-year survival rate of patients with localized OS has reached approximately 75% under multimodality treatment.^[2]^ Nevertheless, metastatic OS, no more than 20%, still faces enormous challenges in increasing the low survival rate.^[3]^ Despite significant advances in multimodality treatment, the survival rate of patients with metastatic OS is just similar as 2 decades ago.^[4]^

Nowadays, increasing evidence indicates that both tumor-associated elements but also on host-associated elements are under a remarkable effect on the prognosis of cancer patients, especially systemic inflammatory response.^[5,6]^ Circulating monocyte count and neutrophil/lymphocyte ratio (NLR), as meaningful indicators inflammation status of patients, was proved to be prognosis predictor in various carcinomas.^[7–13]^ Increasing neutrophils and/or decreasing counts of lymphocytes might suppress lymphocyte-activation, which would be deleterious to prognosis and survival.^[14]^

In OS patients, evaluating the high risk factor of developing metastases would be an important early step. It is necessary to select proper candidates with high risk of developing metastases and carefully evaluates more aggressive measures.^[15]^

Current study was designed to improve the outcomes of the metastases by developing a predictive model considering circulating leukocyte influence to estimate the pretest probability of developing metastases in patients with OS.

## 2 Method

The institutional board of the first affiliated hospital of Anhui Medical University has approved the current study protocol. All patients have signed written informed consents: including tissue analysis and potential therapeutic research.

Medical records from 290 consecutive OS patients between July 2001 and May 2013 were reviewed. Characteristics of patients and tumors at initial diagnosis of OS and development of metastases were collected. Metastases were definitively diagnosed through pathological examinations for tissues obtained via operations or biopsies. The following factors were studied: patient age, gender (male vs female), primary tumor size, tumor location (femur, tibia, humerus, fibula, and others), tumor grade (1–4), and histological classification (osteoblastic, chondroblastic, and others). WHO classification was used for the determination of pathological diagnosis and tumor grade. All pathological diagnoses were established after examining and confirming by 2 independent experienced pathologists.

The calculation of monocyte ratio was the absolute monocyte count after initial treatment divided by the absolute monocyte count before initial treatment. The equation of NLR was described as the neutrophil count divided by lymphocyte count. NLR ratio was calculated as NLR after initial treatment divided by NLR before initial treatment.

### 2.1 Statistical analysis

For categorical data, the Fisher exact test or Pearson χ^2^ test were used. Accordingly, the Mann–Whitney *U* test or independent sample *t* test were employed.

To develop the predictive model, stepwise logistic regression was used, where the final diagnosis was set as the dependent variable and the following characteristics as independent variables: patient age, gender, primary tumor size, tumor location, tumor grade, histological classification, monocyte ratio, and NLR ratio. The final model was established through eliminating variables by backward selection, where the selective criterion was statistically significant level of 0.05. If using a relevantly more liberal *P* value of 0.10, similar results would be observed. After tested all potential clinical interactions, since no statistically significant results were found, all of them were eliminated in the final model. Furthermore, all predictors entered in the final model were reported their odds ratios (ORs) and 95% confidence intervals (CIs).

The final model could be applied to compute the estimated probabilities of metastases for study individuals. To construct the receiver-operating characteristic curve, the predicted probabilities and definitive diagnoses of metastases were used. Then, in order to describe the accuracy of the model, the AUCs and their 95% CI were reported. To estimate model fit, the Hosmer–Lemeshow goodness-of-fit statistic (*P* > 0.05) was used. The cross-validation procedure was selected in model validation procedure, whose advantages were allowing to access the entire dataset for model validation. *P* values < 0.05 was defined as the criterion of statistical significant. Data analysis was performed using IBM SPSS Statistics 22.0 for Windows (SPSS Inc, Chicago, IL).

## 3 Result

A total of 290 patients with OS were taken account in the final analysis. In this group of 290 patients, the mean age was 14 years (median 15 years, range: 5–21 years); 180 patients were female (62.1%) and 110 were male (37.9%). The tumor pathological subtypes included osteoblastic in 131 patients (45.2%), chondroblastic in 68 patients (23.4%), and others in 91 patients (31.4%) (Table 1).

**Table 1:**
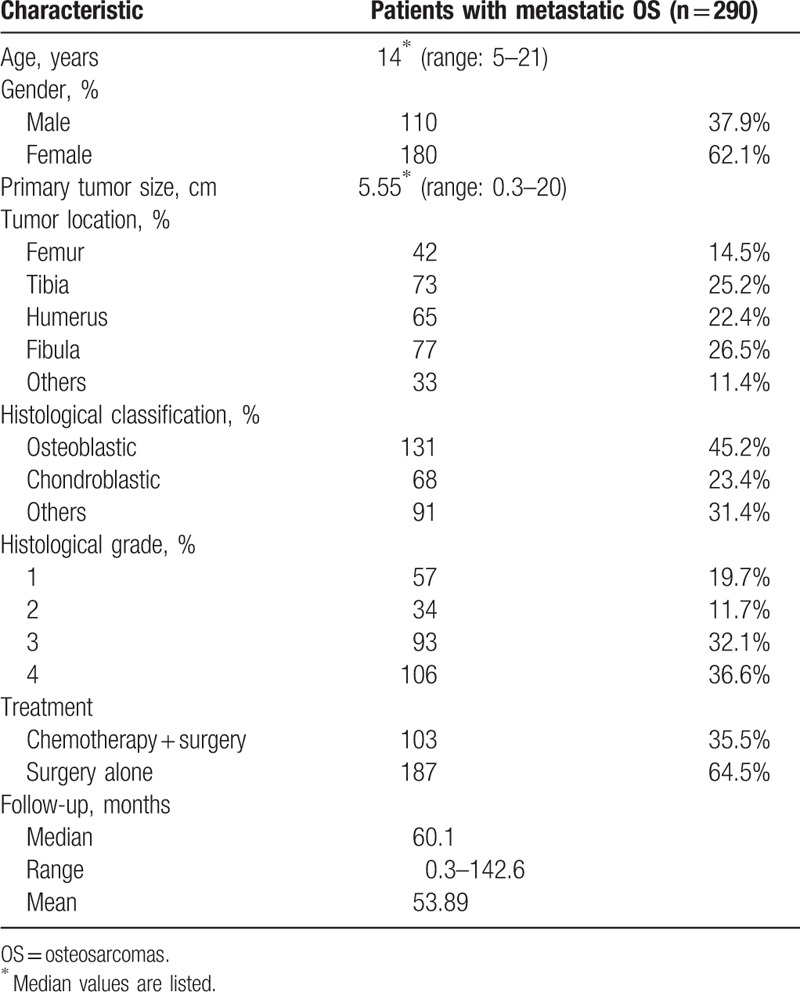
Clinicopathologic characteristics of patients with OS.

As of December 2014, the mean follow-up period of the entire cohort was 53.89 months (median 60.1 months, range: 0.3–142.6 months). The mean tumor size at diagnosis was 6.51 cm (range 0.3–20 cm, median 5.55 cm).

Victims with metastatic OS would establish the following features: higher tumor grade, monocyte ratio >1, and NLR ratio >1. All the 3 features were tested to be statistically significant.

Finally, under multivariate logistic regression analysis, 2 independent variables were identified as predictors of metastases (Table 2). Other potential predictors, since shown not associated with metastases, were excluded in the final model. Individuals with monocyte ratio >1 were over 5 times more likely to develop metastases than patients with monocyte ratio <=1 (OR 5.367; 95% CI, 3.083–9.343). The likelihood of developing metastasis was higher than 4-fold for OS patients with NLR ratio >1 than those with NLR ratio <=1 (OR 4.631; 95% CI, 2.474–8.667).

**Table 2:**
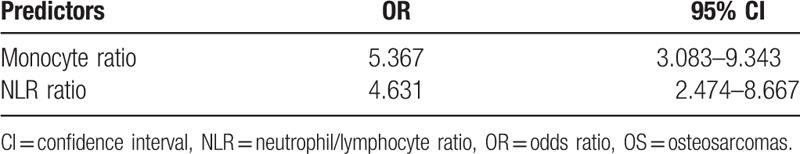
Predictors of metastatic OS.

The final predictive model was demonstrated as this equation: probability of developing metastases = ex/(1 + ex), x = −2.150 +  (1.680 × monocyte ratio)  + (1.533 × NLR ratio). In this equation, e is the base of the natural logarithm, and the assignments of monocyte ratio and NLR ratio are 1 if the ratio >1 (otherwise 0). In addition, the Hosmer–Lemeshow test revealed a *P* value of 0.989, which meant well model fit. Moreover, the result of correlation matrix of parameter estimates illustrated little likelihood of existing multicollinearity.

**Figure 1:**
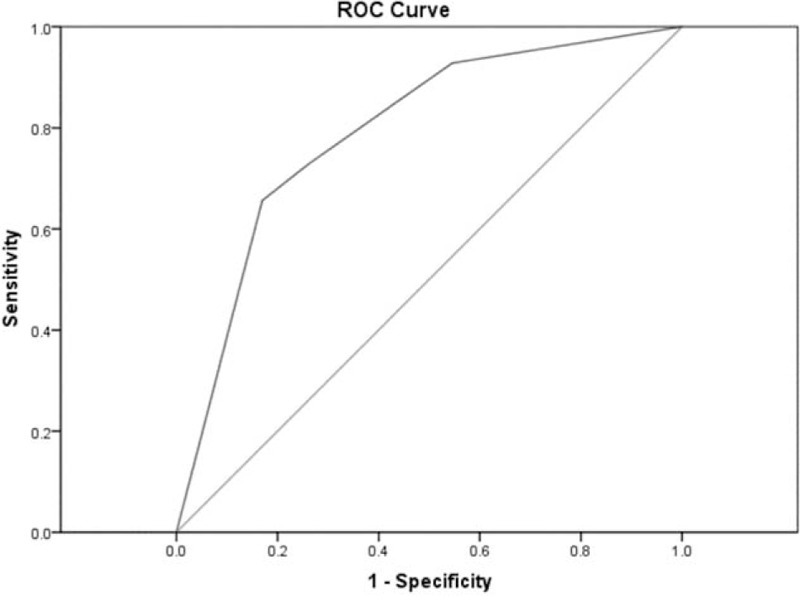
Receiver-operating characteristic (ROC) curve for the clinical prediction model.

The calculated AUC of the receiver-operating characteristic curve as 0.793 revealed well accuracy of this model (95% CI, 0.740–0.845) (Fig. 1). Accordingly, from the cross-validation procedure, a similar AUC as 0.743 was generated (95% CI, 0.684–0.803). Additionally, the result of cross-validation procedure (leave-one-out) demonstrated a substantial agreement (Kappa index of agreement: 0.493) (Table 3).

**Table 3:**
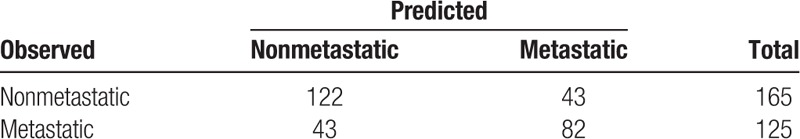
Result of cross-validation procedure (leave-one-out).

## 4 Discussion

Current model summarized a single institutional experience of OS from cancer institution. Despite continuous advances in treatment, the prognosis of OS is still unsatisfying in the past decades if developing metastases.^[16,17]^ This is the reasons that we look for the methods to improve the outcomes of the metastases prediction and select the proper candidates with high risk of developing metastases to carefully evaluate for more aggressive measures.

Previous studies indicated that invasion and metastatic ability were affected by both the intrinsic characteristics and microenvironment of tumor.^[18]^ Moreover, extensive inflammatory response could be triggered and followed by tissue breakdown and disintegration.^[19]^ Evidence has also showed the relationship between prognosis, progressive nutritional decline of cancer patients, and systemic inflammatory response, which might be partly interpreted by insidious cancer aggressiveness activating innate immunity.^[20]^

Decreasing lymphocytes, and increasing monocytes, neutrophils, as consequences of these inflammatory processes, have already been proved to promote inflammation-induced tumor growth through various proangiogenic cytokines, although widely existence of immune suppressive status in cancer population.^[21–25]^

Peripheral blood monocytes have been shown to be an independent prognosis factor in neck and head, gastric, and cholangiocarcinoma cancers.^[26–28]^ Additionally, many investigations have illustrated peripheral blood neutrophils and lymphocytes as prognosis predictors of cancer patients.^[29–31]^ What is more, elevating NLR was associated with poor prognosis in ovarian cancer and gastric cancer by innumerable evidences.^[32,33]^ This association might be due to suppress antitumor immune activity via natural killer cells and lymphocytes. Previous evidences on circulating leukocyte influenced us to pay attention on the significance of clinical circulating monocytes, neutrophils, and lymphocytes. Previous predictive models have been designed to compute the probability of clinical outcomes in other cancers.^[34,35]^ They could estimate the probability of exact clinical outcomes with appropriate accuracy.^[36]^ Differently, relatively large number of patients with OS were enrolled in our study to establish a clinical predicting model on metastases. Furthermore, monocyte and NLR ratio, instead of cells counts, could evaluate the response to treatment, were used in our analysis.

Several limitations remained in this study. First, a relatively small number of clinical predictors were examined in this study, regardless of the fact that the statistical analysis indicates well and sufficient model fit. Second, monocyte phenotype or molecular information was not analyzed, which were caused by lack of this information in our retrospective data.

In the current study, the model could be used to compute pretest probabilities of developing metastases in OS patients, and the equation might also be incorporated into a formal decision analysis. Despite the fact that the accuracy of the model was satisfactory, we accentuate that the model would not be devoted as a stand-alone test, but rather as a tool to guide the selection.
